# Extracorporeal shock wave therapy inhibits osteoclast differentiation by targeting NF-κB signaling pathway

**DOI:** 10.1186/s13018-023-04166-w

**Published:** 2023-10-27

**Authors:** Bei Chen, Yeqiang Luo, Zhongxiu Zhang, Shanghui Lin, Renkai Wang, Baofeng Li

**Affiliations:** 1 Department of Radiation Oncology, Nanfang Hospital, Southern Medical University, Guangzhou, China; 2 Department of Orthopaedics, General Hospital of Southern Theater Command of PLA, Guangdong Key Lab of Orthopedic Technology and Implant Materials, Guangzhou, China; 3 Department of Anesthesiology, General Hospital of Southern Theater Command of PLA, Guangzhou, China

**Keywords:** Extracorporeal shock wave therapy, Osteoclast differentiation, NF-κB signaling pathway, NTAFc1

## Abstract

**Background:**

Extracorporeal shock wave therapy (ESWT) has been reported to promote osteoblast differentiation. However, the role of ESWT on osteoclast differentiation is still elusive.

**Methods:**

This study analyzed the differentiation of osteoclasts in the shock wave group and the control group in vitro, and TRAP staining, RT-PCR, WB assays, and MTT assays were assessed between the two groups. Furthermore, we analyzed the bone formation in these two groups in vivo and micro-CT and trap staining were assessed between the two groups.

**Results:**

We found that ESWT inhibited osteoclast maturation in vitro and ESW treatment of femur promoted bone formation in vivo. Mechanically, osteoclast differentiation was inhibited as the number of impulses increased and ESWT decreased endogenous levels of NTAFc1 and P65 protein.

**Conclusions:**

ESWT may be a potential therapy of osteoporosis through NF-κB signaling pathway.

## Introduction

Osteoporosis is a chronic skeletal disease mainly affecting the elderly and post-menopausal female patients [1], which is manifested as decreased bone density, thinning of bone cortex, widening of bone marrow cavity, decreased number of bone trabeculae, and prone to fractures [2]. In the osteoporosis microenvironment, bone marrow mesenchymal stem cells (BMSCs) are lacking and osteoclasts are overactive, which leads to insufficient bone formation and excessive bone resorption, resulting in excessive bone loss [3]. With the aging of the population, the prevalence of osteoporosis and the incidence of fragility fracture have been increasing in recent years. In addition, the treatment of severe bone defects caused by high-energy trauma, infection, and tumor resection is one of the most challenging clinical problems facing orthopedic surgeons [4]. Therefore, the study of effective strategies to promote the healing of key bone defects in osteoporosis has important clinical significance to solve these problems.

Extracorporeal shockwaves (ESW), a kind of rapid oscillation of pressure wave that can propagate through various media [5]. Previously, studies have revealed that the application of ESWT upregulates angiogenesis and growth factors by activating endothelial nitric oxide synthase (eNOS) and vascular endothelial growth factor (VEGF) [6]. The increase in the formation of nitric oxide caused by eNOS activation promotes the differentiation of human osteoblasts [7]. Furthermore, Extracorporeal shock wave can treat bone tissue and skeletal muscle related tissue injury [8], including knee Contracture [9], bone nonunion [10], and tendon injury [11]. we also found that ESWT could promoted osteogenic differentiation in a rabbit osteoporosis model through TGF-β/SMAD2 pathway [5]. However, the effect of ESW treatment on osteoclast differentiation remains to be elucidated in osteoporosis.

In our study, we cultured RAW264.7 cells treated with ESW and differentiated them into osteoclast to determine the roles of ESWT in osteoclast differentiation and the potential mechanism. Furthermore, we generated osteoporosis mice to illustrate the therapeutic effect of ESWT, which provided an experimental basis for ESWT to treat osteoporosis clinically.

## Methods and materials

### Cell culture

RAW264.7 cells (ATCC, USA) were cultured in high glucose DMEM (4.5 g/100 ml) (Gibco, USA) supplemented with 10% fetal bovine serum (FBS) (Cellmax, China) and 100 U/mL penicillin (Gibco, USA) and 100 μg/mL streptomycin (Gibco, USA).

For the induction of osteoclastogenic differentiation, we seeded RAW264.7cells into each well of 24-well plates at a density of 1000 cells/cm^2^ and then administrated them with 100 ng/mL receptor activator for nuclear factor κB ligand (RANKL; PeproTech, USA) and 30 ng/mL recombinant murine macrophage colony-stimulating factor (M-CSF; PeproTech, USA) for 7 days.

### Trap staining

We used a commercial TRAP kit (Sigma-Aldrich, USA) to identified osteoclast cells. Briefly, we fixed cells with 4% paraformaldehyde for 30 min. Then, we washed three times with 1X PBS, cells were incubated with TRAP staining buffer according to the general protocols. TRAP-positive cells (more than three nucleus) can be identified as osteoclast cells, respectively.

### MTT assays

MTT assays were performed as previously described [5]. Briefly, we cultured RAW264.7 cells treated with ESW therapy in 24-well plates at a density of 1000 cells/cm^2^. Then, 40 ml of MTT was added into each well at the corresponding time point for 6 h, and then discarded the liquid and added 150 μl of DMSO, shake for 15 min. OD value of 495 nm wavelength was detected in the enzyme-linked immunoassay instrument.

### Western blotting

Western blotting was performed as previously described [12]. Briefly, we separated total cell lysates by SDS-PAGE and blotted on the membranes (CST, USA). These membranes were incubated with specific antibodies to NFATc1(1:1000, Abcam), P65 (1:1000, CST), and GAPDH (1:2000, CST), then we reprobed them with appropriate horseradish peroxidase-conjugated secondary antibodies. Blots were visualized by enhanced chemiluminescence (ECL Kit; CST).

### Real-time PCR

We performed qRT-PCR analysis by using a Roche Molecular Light Cycler (Roche) as previously described [13]. We isolated Total RNA by using total Trizol reagent (Invitrogen). Then, we performed reverse transcription by using 1 mg total RNA and Super-Script II (Invitrogen). And, set up amplification reactions in 50 μl reaction volumes containing SYBR Green PCR Master Mix (PE Applied Biosystems), 2 μl volume of cDNA, and amplification primers. The primer used for real-time PCR are listed in Table 1.

**Table 1:** Primers list

Gene name		Sequence 5′-3′	Length	Tm	Accession
Gapdh	Forward primer 5′-3′	AAT GGA TTT GGA CGC ATT GGT	21	60.6	PrimerBank ID 6679938c1
	Reverse primer 5′-3′	TTT GCA CTG GTA CGT GTT GAT	21	60.2	
Ctsk	Forward primer 5′-3′	CTC GGC GTT TAA TTT GGG AGA	21	60.4	PrimerBank ID 142352209c1
	Reverse primer 5′-3′	TCG AGA GGG AGG TAT TCT GAG T	22	61.2	
DC-STAMP	Forward primer 5′-3′	AAG GTG GTG GCG TTA TAC TGC	21	62.4	PrimerBank ID 116812882c1
	Reverse primer 5′-3′	CTG GCA CAG CGG ATG TGA G	19	63.0	

### Mice and ESWT treatment

For ESW therapy, we used the shock wave treatment machine (EposUltra, Dornier MedTech, Wessling, Germany) for ESW treatment experiment as previously described [5]. This shock wave generator was an electromagnetic pulse type shock wave generator, and we set the selectable energy flow density range from 0.03 to 0.5 mJ/mm2 and the impulses range from 500 to 2000 impulses. The anesthetized mice lied on its side on the treatment table, and we cut off the experimental site hair. Then, the experimental femoral condyles of the grouped mice were subjected to ESW treatment every 3 days for 4 weeks with energy flow density of 0.12 mJ/mm^2^, pulse 2000 times, frequency 4 Hz, focal length (penetration depth) 10 mm, and no treatment was done on the left side as a self-control. The mice were sacrificed at 4 weeks after the operation.

### μCT analysis

We scanned femur samples dissected from mice and fixed for 24 h with 4% paraformaldehyde, then scanned and analyzed with micro-CT (Quantum GX, PE). Micro-CT scans were performed under the same conditions: voltage 80 kV, current 80 mA, spatial resolution 14 mm, scanning 500 continuous sections [3]. Then, we analyzed the data through computer software to collect the number of trabecular bones (Tb.N), trabecular bone thickness (Tb.Th), trabecular bone space (Tb.Sp), bone volume fraction (BV/TV), and other indicators.

### Statistical analysis

All the data were exhibited as the mean ± standard deviation (S.D.). Two groups were analyzed by Student’s t-test, and multiple groups were assessed using one-way analysis of variance (ANOVA). When *P* < 0.05, the data was considered statistically significant.

## Results

### ESWT inhibited osteoclast differentiation in vitro.

**Fig. 1:**
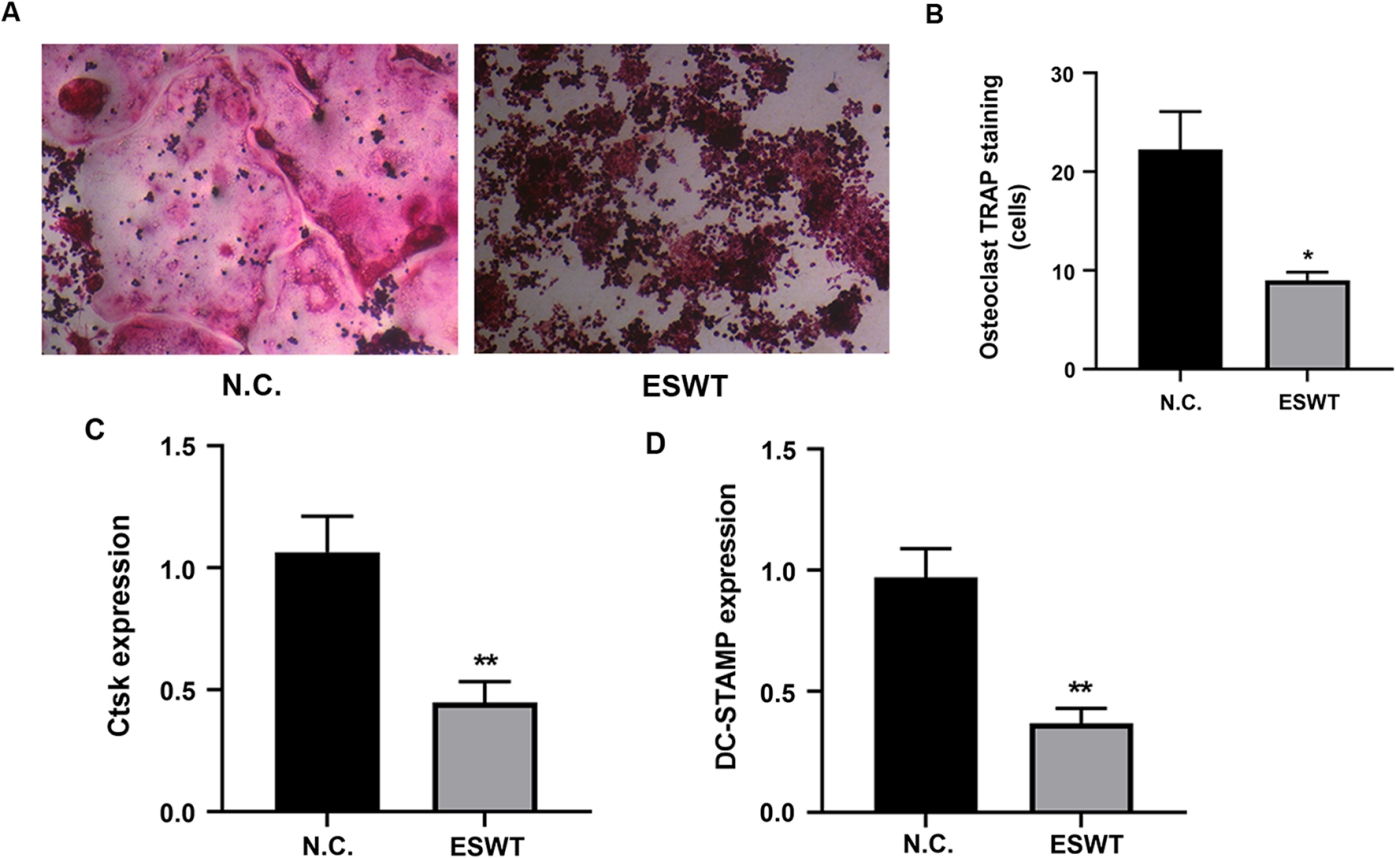
ESWT inhibited osteoclast differentiation in vitro. **A** The TRAP staining of RAW264.7 cells cultured in DMEM treated with N.C. or ESW treatment. Scale bar: 100 μm. **B** Quantification of TRAP-positive cells, *n* = 4 per group. **C** The relative mRNA expression of Ctsk and DC-STAMP of RAW264.7 cells cultured in DMEM treated with N.C. or ESW treatment after 48 h. *P < 0.05; **P < 0.01

To examine the role of ESWT on the osteoclast differentiation of RAW264.7 cells, TRAP staining was performed and found that RAW264.7 cells treated with ESW therapy exhibited TRAP-positive osteoclasts significantly (Fig. 1A, B ). Furthermore, compared with negative control, ESW therapy caused significant decreased expressions in osteoclast differentiation related markers of Ctsk and DC-STAMP (Fig. 1C). Thus, these data suggest that osteoclast differentiation was inhibited by ESW therapy.

### ESWT increased bone formation in vivo.

**Fig. 2:**
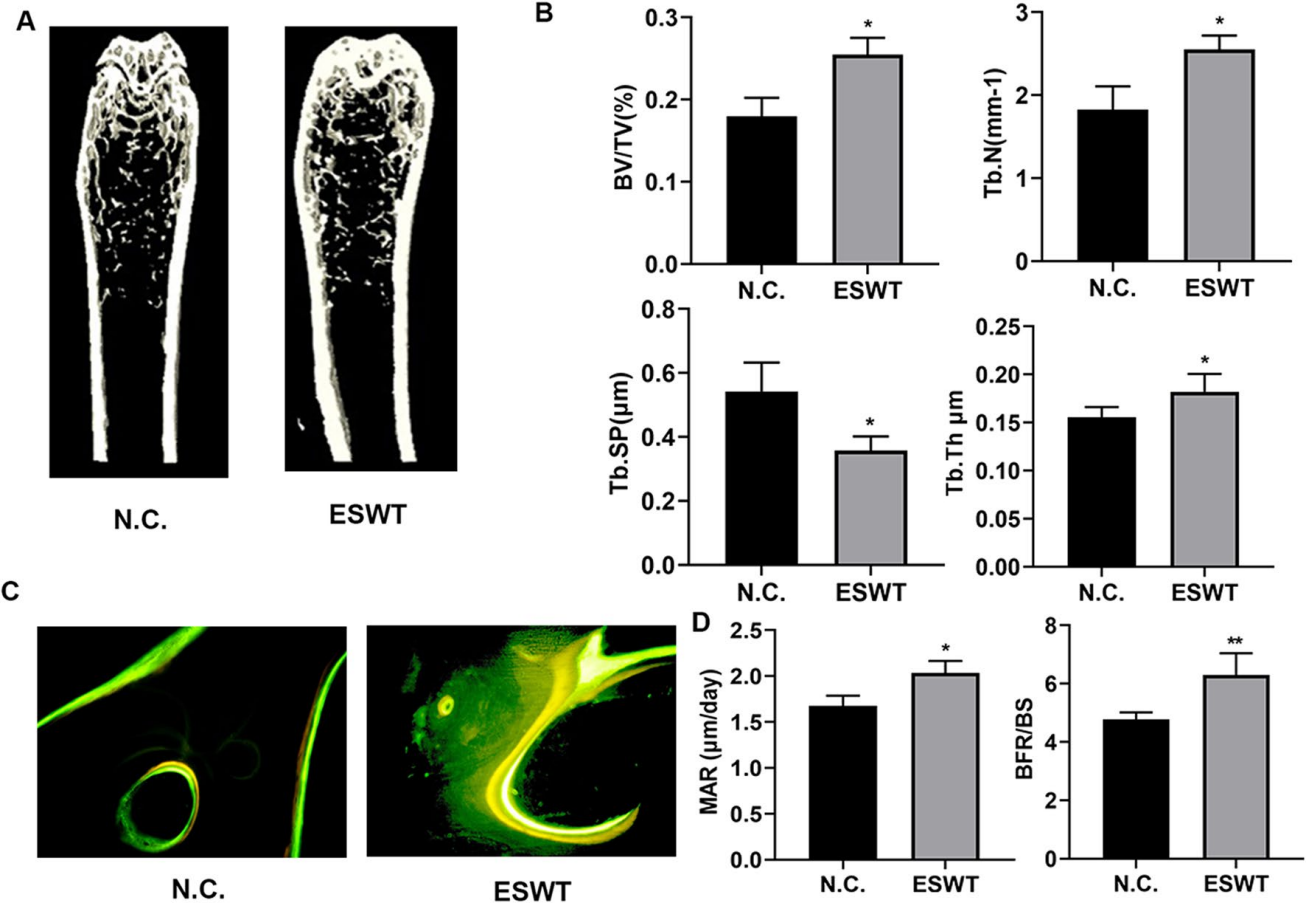
ESWT increased bone formation in vivo. **A** Representative μCT images of femora from mice treated with ESW or N.C.. Scale bar: 100 mm. **B** Quantitative μCT analysis of femora. **C** Representative images of calcein (green) and tetracycline (yellow) labeling of trabecular bone (**D**) with quantification of mineral apposition rate (MAR) and quantification of the BFR per bone surface (BFR/BS) of femora. Scale bar: 50 μm. *n* = 6, per group. Data are reported as the mean ± S.D. *P < 0.05; **P < 0.01

To test the effects of ESW therapy in vivo, ESWT was treated in mice femora. μCT revealed that increased trabecular bone volume (BV/TV%), number (Tb.N), and thickness (Tb.Th), and decreased trabecular separation (Tb.Sp) in mice femurs compared with those negative controls (Fig. 2A, B). Furthermore, calcein (Green) and tetracycline (yellow) double labeling indicated that ESW therapy group had significantly increased mineral apposition rates (MAR) and the quantification of the BFR per bone surface compared with those negative controls (Fig. 2C, D). In a conclusion, these results showed that ESWT inhibited osteoclast differentiation in vivo.

### ESWT inhibited RAW 264.7 proliferation by targeting NF-κB signaling.

**Fig. 3:**
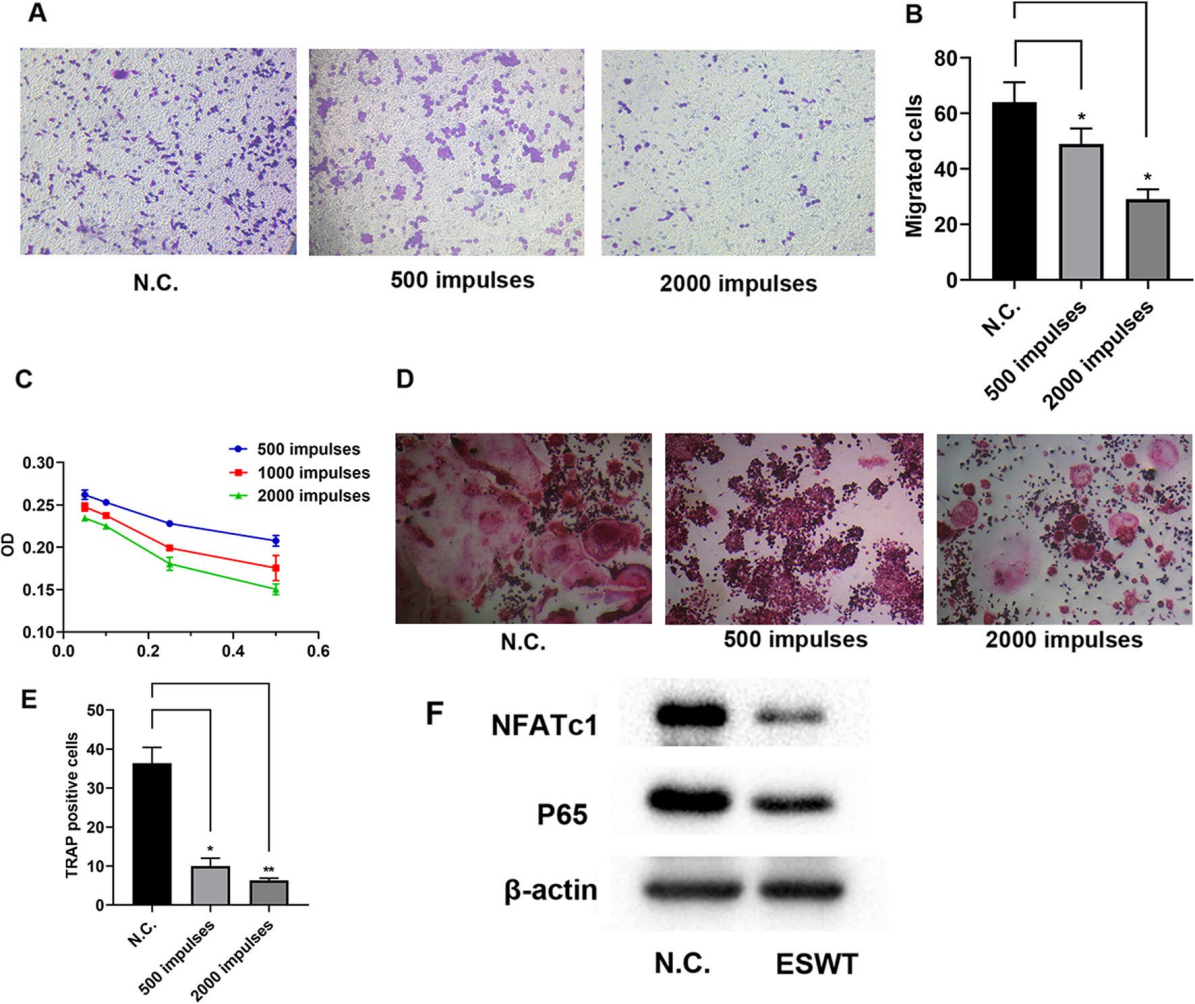
ESWT inhibited RAW 264.7 proliferation by targeting NF-κB signaling. **A** RAW264.7 cells motility in different impulses treatment groups were evaluated by the transwell migration assays and quantitative analysis of migrated cells of them (**B**) (Scale bar: 100 μm). **C** MTT analysis of RAW264.7 cells proliferation in different treatment groups. *n* = 4 per group. **D** The TRAP staining of RAW264.7 cells cultured in DMEM treated with N.C., 500 impulses and 2000 impulses. **E** Quantification of TRAP + cells, *n* = 5 per group. **F** WB analysis of NFATc1 and P65 in RAW264.7 cells treated with ESW or N.C

To investigate the influence of different energy density or impulse times on osteoclastic differentiation, different energy density or impulse times were performed on RAW264.7 cells and revealed that the migration of RAW264.7 cells was decreased as the number of impulses increased (Fig. 3A, B). Furthermore, MTT assays showed that the survival rate of RAW264.7 cells was inhibited as the energy flow density or the number of impulses increased (Fig. 3C). TRAP staining also revealed that high frequency impulses inhibited osteoclast differentiation compared those deal with low frequency impulses (Fig. 3D, E). Mechanically, NFATc1 has been reported to associate with osteoclast differentiation and promote bone resorption by targeting NF-κB pathway. Here, we found that ESWT inhibited endogenous levels of NFATc1 and P65 protein expression (Fig. 3F). In total, these data revealed that osteoclast differentiation was inhibited as the number of impulses increased and ESWT inhibited RAW 264.7 proliferation by targeting NF-κB signaling.

### ESWT might be a potential therapy for osteoporosis

**Fig. 4:**
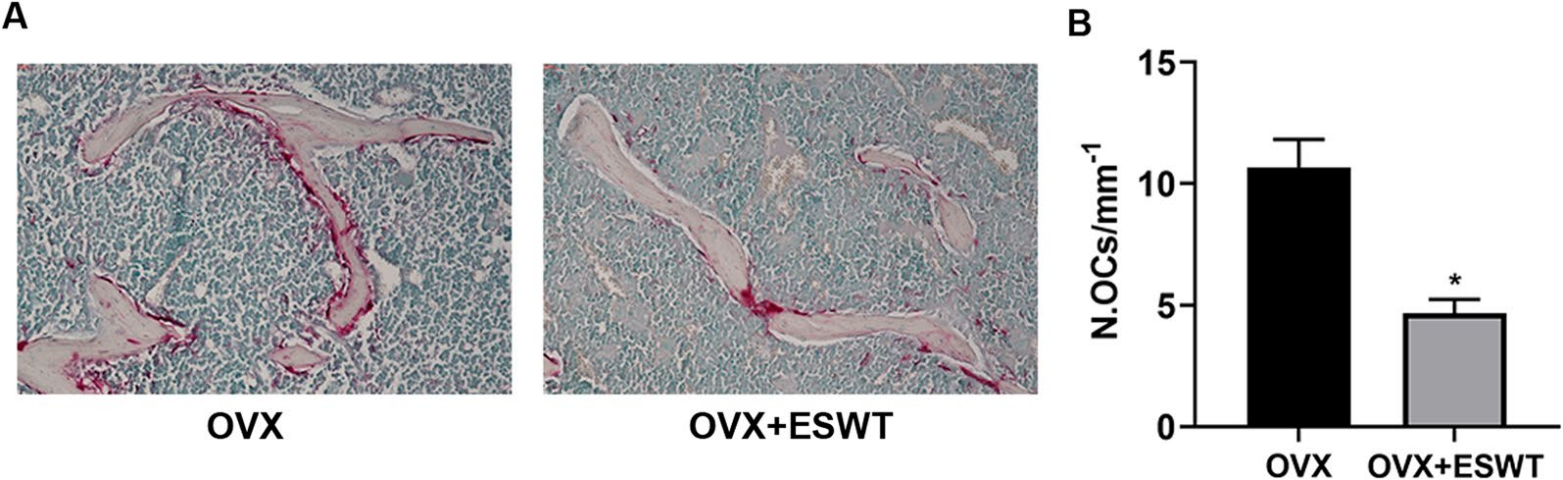
ESWT might be a potential therapy for osteoporosis. **A** Representative images of TRAP staining in mice femora treated with ESW or N.C. **B** Quantitative analysis of the number of osteoclasts (OCs) on trabecular bone surface in **A**. *n* = 7 per group

**Fig. 5:**
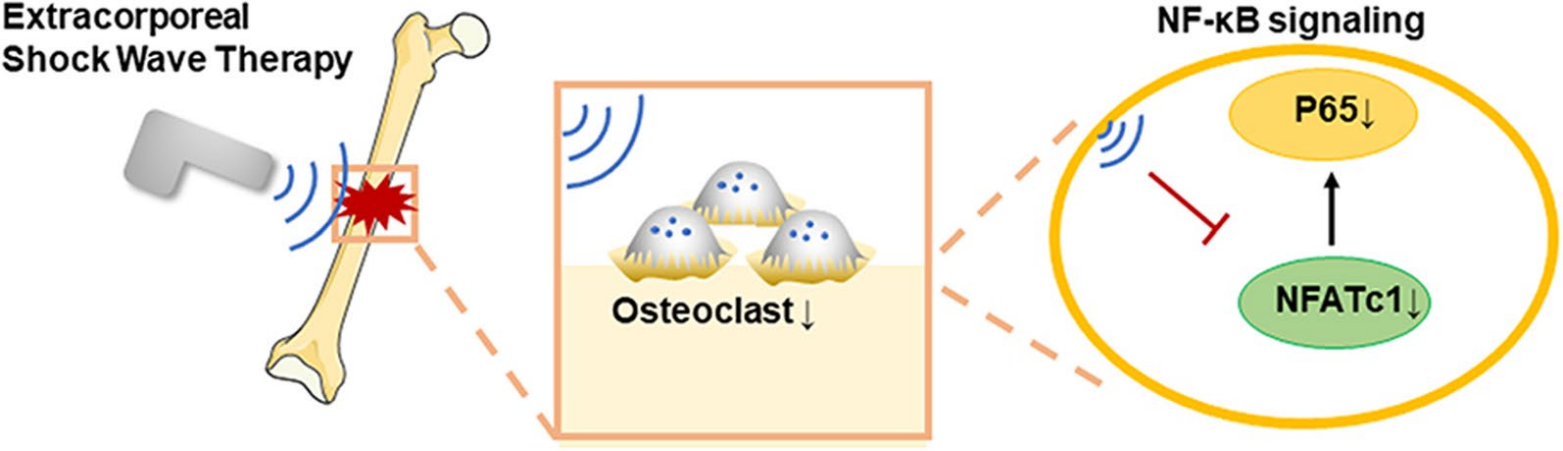
Schematic of the working hypothesis. ESWT can promote local bone formation by inhibition of osteoclast differentiation. Furthermore, ESWT inhibited NFATc1 and P65 expression in vitro. In a conclusion, ESWT inhibits osteoclast differentiation by targeting NF-κB signaling pathway

To investigate the therapeutic potential of ESW therapy on osteoporosis. We generated mice osteoporosis model and TRAP staining showed that osteoclast maturation was inhibited in vivo (Fig. 4A, B). In conclusion, these results show that ESW therapy inhibited bone resorption and prevented bone loss on osteoporosis through NF-κB signaling pathway (Fig. 5).

## Discussion

In this study, we found that ESWT inhibited osteoclast differentiation in vitro and decreased the expression of Ctsk and DC-STAMP. Furthermore, ESWT could increase bone formation in vivo. Moreover, as the energy flow density or the number of impulses increased, the proliferation of RAW264.7 cells was decreased and the osteoclast differentiation was inhibited. In addition, ESW treatment in vivo could ameliorated bone loss and osteoporosis.

Previously, it has reported that the applications of ESWT in the treatment of bone disorders have grown to include in non-union or delayed union of fracture, osteoporosis, spinal cord injury, ervical spondylosis, and scoliosis [14]. The bone formation effect of extracorporeal shock wave therapy is manifested in the following aspects: (1) Mechanism and chemical transduction: Stress stimulation plays an important regulatory role in bone growth, absorption, and reconstruction. As a physical signal, ESW will generate tensile stress, compressive stress, and shear stress between the interfaces and the surface of tissue cells when it is conducted between different media. Osteoblasts, by sensing mechanical stimuli and converting biological stimuli into biochemical signals, further affect cell gene expression, activate bone marrow stromal stem cells, promote the proliferation of osteoblasts, and play an important role in bone regeneration [15]. (2) Cavitation effect: When the shock wave is transmitted in the human tissue, because the tissue contains small bubbles, the gas will expand at a very fast speed under the condition of sudden pressure changes and produce higher energy in a small range. This effect can release adhesions and improve local blood circulation, thereby achieving a therapeutic effect [16]. (3) The sealing effect of pain sensory nerve receptors: The shock wave stimulates the pain sensory nerve receptors, which changes the receptor's receiving frequency of pain and the composition of the surrounding chemical mediators, inhibits nerve ending cells and prevents subsequent centripetal impulses from being transmitted, thus alleviating local pain [17]. In our study, we mainly elaborated ESWT inhibited osteoclast differentiation in vitro and decreased endogenous levels of NTAFc1 and P65 protein which provides a theoretical basis for ESWT to treat osteolysis-related diseases. Our work might provoke interesting future works in osteolysis and ESWT.

Furthermore, ESW has the effects of promoting tissue metabolism, relieving pain and promoting tissue repair. It also shows good curative effects in the treatment of some chronic soft tissue diseases. Ogden et al. reported that ESW has a total effective rate of up to 88% in the treatment of plantar fasciitis, 80% of patients only need one treatment, and 20% of patients only need two treatments [18]. Chen et al. treated 80 patients with heel pain. After 6 months, 59.3% had pain disappeared, 27.7% had obvious improvement, and 13% had mild improvement [19]. Rompe et al. used ESW and traditional surgical methods to treat calcified tendinitis of the shoulder. The excellent and good rate of ESW reached 60% after 1 year and 64% after 2 years, which is better than traditional surgical treatment [20].

## Conclusions

The present study revealed that ESWT inhibited osteoclast differentiation, enhanced the quantity and quality of local cortical bone in osteoporosis mice models. The negative effects of ESWT on proliferation and osteoclastic differentiation of RAW264.7 cells may be an important mechanism. These results could provide theoretical basis for the prevention and treatment of osteoporosis.

## Data Availability

The datasets analyzed during the current study are not publicly available due privacy but are available from the corresponding author on reasonable request.

## Abbreviations

ESWT: Extracorporeal shock wave therapy
ESW: Extracorporeal shock wave
FBS: Fetal bovine serum
RANKL: Receptor activator for nuclear factor κB ligand
M-CSF: Macrophage colony-stimulating factor
TRAP: Tartrate-resistant acid phosphatase
Ctsk: Cathepsin K
NF-κB: Nuclear factor kappa B
NFATc1: Nuclear factor of activated T-cells
BMSCs: Bone marrow mesenchymal stem cells

## References

[1] Xu SY, Shi P, Zhou RM (2021). Post-menopausal oestrogen deficiency induces osteoblast apoptosis via regulating HOTAIR/miRNA-138 signalling and suppressing TIMP1 expression. J Cell Mol Med.

[2] Clark D, Nakamura M, Miclau T, Marcucio R (2017). Effects of aging on fracture healing. Curr Osteoporos Rep.

[3] Wang R, Zhang H, Ding W, Fan Z, Ji B, Ding C (2020). miR-143 promotes angiogenesis and osteoblast differentiation by targeting HDAC7. Cell Death Dis.

[4] Loi F, Cordova LA, Pajarinen J, Lin TH, Yao Z, Goodman SB (2016). Inflammation, fracture and bone repair. Bone.

[5] Li B, Wang R, Huang X, Ou Y, Jia Z, Lin S (2021). Extracorporeal shock wave therapy promotes osteogenic differentiation in a rabbit osteoporosis model. Front Endocrinol (Lausanne).

[6] Schleusser S, Song J, Stang FH, Mailaender P, Kraemer R, Kisch T (2020). Blood flow in the scaphoid is improved by focused extracorporeal shock wave therapy. Clin Orthop Relat Res.

[7] Fiani B, Davati C, Griepp DW, Lee J, Pennington E, Moawad CM (2020). Enhanced spinal therapy: extracorporeal shock wave therapy for the spine. Cureus.

[8] Maffulli G, Hemmings S, Maffulli N (2014). Assessment of the effectiveness of extracorporeal shock wave therapy (ESWT) for soft tissue injuries (ASSERT): an online database protocol. Transl Med UniSa.

[9] Maffulli N (2023). CORR insights(R): extracorporeal shock wave therapy improves nontraumatic knee contracture in a rat model. Clin Orthop Relat Res.

[10] Wu W, Maffulli N, Furia JP, Meindlhumer L, Sternecker K, Milz S (2021). Exposure of zebra mussels to radial extracorporeal shock waves: implications for treatment of fracture nonunions. J Orthop Surg Res.

[11] Schmitz C, Csaszar NB, Milz S, Schieker M, Maffulli N, Rompe JD (2015). Efficacy and safety of extracorporeal shock wave therapy for orthopedic conditions: a systematic review on studies listed in the PEDro database. Br Med Bull.

[12] Zhang H, Wang R, Wang G, Zhang B, Wang C, Li D (2021). Single-cell RNA sequencing reveals B cells are important regulators in fracture healing. Front Endocrinol (Lausanne).

[13] Huang J, Yin H, Rao SS, Xie PL, Cao X, Rao T (2018). Harmine enhances type H vessel formation and prevents bone loss in ovariectomized mice. Theranostics.

[14] Shi L, Gao F, Sun W, Wang B, Guo W, Cheng L (2017). Short-term effects of extracorporeal shock wave therapy on bone mineral density in postmenopausal osteoporotic patients. Osteoporos Int.

[15] Wang FS, Wang CJ, Huang HJ, Chung H, Chen RF, Yang KD (2001). Physical shock wave mediates membrane hyperpolarization and Ras activation for osteogenesis in human bone marrow stromal cells. Biochem Biophys Res Commun.

[16] Roehrig GJ, Baumhauer J, DiGiovanni BF, Flemister AS (2005). The role of extracorporeal shock wave on plantar fasciitis. Foot Ankle Clin.

[17] Hsu RW, Hsu WH, Tai CL, Lee KF (2004). Effect of shock-wave therapy on patellar tendinopathy in a rabbit model. J Orthop Res.

[18] Ogden JA, Alvarez R, Levitt R, Cross GL, Marlow M (2001). Shock wave therapy for chronic proximal plantar fasciitis. Clin Orthop Relat Res.

[19] Chen HS, Chen LM, Huang TW (2001). Treatment of painful heel syndrome with shock waves. Clin Orthop Relat Res.

[20] Rompe JD, Zoellner J, Nafe B (2001). Shock wave therapy versus conventional surgery in the treatment of calcifying tendinitis of the shoulder. Clin Orthop Relat Res.

